# The Role of Germline Transposable Element Insertions in Pediatric Cancer Predisposition

**DOI:** 10.1158/2767-9764.CRC-26-0229

**Published:** 2026-09-22

**Authors:** Corinne E. Sexton, Kayla V. Hamilton, Chong Chu, David T. Ting, Judy E. Garber, Peter J. Park, Junne Kamihara

**Affiliations:** 1 Department of Biomedical Informatics, Harvard Medical School, Boston, Massachusetts.; 2 Department of Pediatric Oncology, Dana-Farber Cancer Institute, Harvard Medical School, Boston, Massachusetts.; 3 Division of Hematology/Oncology, Boston Children's Hospital, Boston, Massachusetts.; 4 Division of Cancer Genetics and Prevention, Dana-Farber Cancer Institute, Boston, Massachusetts.; 5 The Wistar Institute, Philadelphia, Pennsylvania.; 6 Krantz Family Center for Cancer Research, Mass General Brigham Cancer Institute, Harvard Medical School, Boston, Massachusetts.; 7 Department of Medical Oncology, Dana-Farber Cancer Institute, Harvard Medical School, Boston, Massachusetts.

## Abstract

At least 10 to 15% of children with cancer have a pathogenic germline variant in a cancer predisposition gene. Studies of germline cancer predisposition, however, have focused primarily on single-nucleotide and copy-number variants, leaving other classes such as transposable elements (TE) largely unexplored. Although rare pathogenic TE insertions have been implicated in inherited cancer, their contribution to pediatric cancer predisposition remains unknown, partly because these repetitive sequences often require whole-genome sequencing (WGS) for detection. We characterized the germline TE insertion landscape using nontumor WGS data from 2,334 patients with pediatric cancer and 3,447 controls. Across 5,781 genomes, we identified 96,484 TE insertions, most of which were rare and located in intergenic or intronic regions. Whereas global TE burden did not differ between cases and controls, rare TE insertions were significantly enriched in cancer genes in patients with solid tumors, particularly within 3′ untranslated regions (*P* < 0.02). Gene–phenotype concordance analysis identified 19 insertions in genes with established dominant cancer predisposition, representing ∼0.8% of cases. Integration of RNA sequencing data revealed transcriptional impact for a subset of insertions. Notably, a 3′ untranslated region LINE-1 insertion in the tumor-suppressor gene *PTEN* disrupted alternative polyadenylation, whereas an SVA insertion in a *STIM1* intron induced exonization, generating a novel transcript containing SVA sequence. These findings demonstrate that rare germline TE insertions in cancer predisposition genes can have functional consequences at the RNA level. Incorporating TE detection into genomic workflows may improve identification of cancer predisposition syndromes and expand understanding of noncoding contributions to pediatric cancer susceptibility.

**Significance:**

At least 10 to 15% of children with cancer have a germline predisposition; however, the true prevalence is likely higher. This study demonstrates that TE insertions can disrupt cancer predisposition genes and have functional consequences. Incorporating TE detection can identify cancer predisposition in children for whom standard testing is uninformative.

## Introduction

Identifying a cancer predisposition syndrome enables individualized assessment of cancer risk and plays a crucial role in early tumor detection and, in some cases, preventive interventions. Pediatric cancers are of particular interest in the study of heritable factors because there is little time for environmentally driven mutations to accumulate and because of the opportunity for early detection and cancer interception. Currently, clinical genetic testing can identify a germline pathogenic or likely pathogenic variant in at least 10% to 15% of children with cancer (1, 2). Many patients with pediatric cancer have personal and family histories highly suggestive of an underlying genetic risk, yet extensive clinical genetic testing fails to identify a causative variant. This limitation is due, in part, to our incomplete understanding of germline predisposition, which has largely been derived from studies of pathogenic variants in the coding regions of cancer predisposition genes. As a result, we lack a full appreciation of germline risk arising from noncoding and repetitive regions of the genome, leading to an inability to identify the full spectrum of hereditary cancer predisposition and limiting opportunities for tailored care and surveillance for affected families (3).

Transposable elements (TE) represent a largely unexplored potential source of germline risk. TEs are repetitive genomic sequences characterized by their ability to self-replicate within the genome. Although the vast majority of TEs are no longer active, a few young subfamilies are still active in humans—namely L1HS, SVA, and Alu elements—via L1HS-mediated retrotransposition mechanisms (4). Each individual genome contains on average more than 1,000 nonreference germline TE insertions (5). Whereas most of these insertions have unknown functional significance, several studies have shown that rare novel germline insertions can be pathogenic. A family with hereditary retinoblastoma for example, with initial uninformative genetic testing, was found to have familial intraocular disease due to a LINE-1 (L1) element inserted into an intron–exon boundary leading to aberrant splicing (6). This L1 insertion resulted in bilateral retinoblastoma in a child and parent and was absent in unaffected family members. Additional pathogenic germline TE insertions in both introns and exons have been found in other cases of retinoblastoma (L1; ref. 7), colon cancer (L1, Alu; ref. 8), and breast cancer (SVA, Alu; refs. 9–11), as well as in Lynch syndrome (Alu, SVA; refs. 12–14) and Gorlin syndrome (Alu; ref. 15). These studies highlight the clinical impact of identifying TEs in patients with cancer and/or suspected cancer predisposition syndromes and the importance of exploring TEs in a broad cohort of pediatric oncology patients.

Because most TE insertions occur outside the coding region, whole-genome sequencing (WGS) is necessary for their detection (16). Moreover, due to their repetitive nature, identification of TE insertions from standard short-read sequencing data requires specialized algorithms. As a result, TEs have not been examined as part of clinical cancer genetic testing, and their role in cancer predisposition remains largely unexplored. To examine the contribution of TEs to pediatric cancer predisposition, we identified TEs from germline WGS data of more than 2,000 patients with pediatric cancer with a broad range of cancer diagnoses. We characterized the prevalence and genomic distribution of TE insertions, showed examples of insertion functional effects in available RNA sequencing (RNA-seq), and demonstrated that, although rare, germline TE insertions represent a substantive source of variation in the study of pediatric cancer predisposition.

## Materials and Methods

### Data and metadata

WGS CRAM and BAM files were obtained from the KidsFirst data portal (including both cancer and birth defect cohorts) and the PROfile And Cancer gene Testing for IndiVidual Evaluation (PROACTIVE) study totaling 6,546 samples (Supplementary Table S1). The cohort comprised 1,734 male and 1,713 female controls and 826 male and 1,508 female probands; among probands with age at phenotype data available (*n* = 4,179), the mean age was 8 years (IQR: 3–13 years). The PROACTIVE study is a Dana-Farber Cancer Institute (DFCI)-wide study that includes germline WGS and RNA-seq of the cohort. Pediatric participants in PROACTIVE were included in this study. The PROACTIVE study and use of the data for this study was approved by the DFCI Institutional Review Board. Written informed consent was obtained from all participants ages 18 years and older and from a parent or legal guardian for participants younger than 18 years. Studies were conducted in accordance with the US Common Rule. dbGAP accession numbers of KidsFirst data included in this study are listed in Supplementary Table S1. Genomes were sequenced from either blood or saliva depending on the study. For the KF-TALL study, blood samples were sorted and enriched for normal mononuclear cells to minimize leukemic contamination and isolate germline DNA (17). All genomes were previously aligned to hg38. Ancestry for PROACTIVE and KidsFirst samples was called by AEon, which uses a 1000 Genomes–generated panel of variants to provide ancestry estimates (bioRxiv 2024.06.18.599246). The population with the largest estimated ancestry fraction was used as the sample’s population throughout the analysis.

### Calling TE insertions

To generate xTea calls, we ran xTea in germline mode with default parameters on all WGS data. More detail can be found at https://github.com/parklab/KidsFirst_TE_pediatric_cancer. Insertions were then merged across the entire cohort if they were from the same TE family (Alu, SVA, and L1) and fell within 35 bp of each other.

### Sample filtering

Samples failing any of the following four criteria were removed from all subsequent analyses:

1. Samples with reported sex must match sex predictions based on X and Y copy-number estimates from indexcov (18).
2. Median genome coverage ≥25.
3. Common Alu insertion recall score >0.83, which corresponds to identifying at least 10 of 12 of near-universal Alu insertions (see below).
4. No more than 60% of 16 kb genome bins with normalized coverage outside of the (0.85, 1.15) interval, as calculated by indexcov (see below; ref. 18).

Applying these metrics filtered 765 of 6,546 samples (11.7% of samples; Supplementary Table S1). Metrics are visualized in Supplementary Fig. S1, and total TE calls which pass quality filters are found in Supplementary Fig. S2.

#### Common Alu insertion recall score

To identify samples with high false negative calls, we extracted Alu insertions with high population allele frequency (AF) in both 1000 Genomes (RRID: SCR_006828; ref. 19) and gnomAD-SV (RRID: SCR_014964; ref. 20) databases. In total, 12 Alu insertions had a population AF > 0.95 and were subsequently used to generate TE insertion recall scores for every sample. The distribution of the common Alu insertion recall score per population is shown in Supplementary Fig. S1.

#### Normalized coverage bins

Because xTea defines a minimum cutoff for insertion supporting reads based on genome-wide coverage estimates, samples with highly variable coverage across the genome can result in extreme false positive calls. To systematically quantify the variation in coverage across the genome, we ran indexcov (18) to obtain normalized coverage estimates for every 16 kb genomic window. Coverage values equal to the median will have a normalized coverage value of 1. To filter our samples, we removed any sample with >60% of 16 kb windows having a normalized coverage value less than 0.85 or greater than 1.15. The distribution of the percent of 16 kb windows outside of (0.85, 1.15) per sample is shown in Supplementary Fig. S1.

### TE insertion annotation

TEs were annotated with a custom script which overlapped GENCODE v45 annotations (with introns and splice sites added by the pyranges library; RRID: SCR_014966) and TE insertions with a slack of 35 bp (see GitHub). Annotations with duplicate features were then prioritized based on the following order: transcription start site, start codon, stop codon, five_prime_UTR, three_prime_UTR, exon, splice site, and intron. For example, an insertion overlapping a splice site and intron would be characterized as a splice site insertion.

### Annotating cancer concordance

Two independent reviewers with expertise in cancer predisposition (K.V. Hamilton and J. Kamihara) reviewed the genes with TE insertions and cancer types of participants to evaluate for concordance. The reviewers based classifications on standard-of-care genetic testing practices and additional literature searches and independently annotated each gene/cancer type as concordant, discordant, or preliminary evidence. The two reviewers’ lists were then compared, and the reviewers met to reconcile any discrepancies. Agreement was reached on all classifications.

### Gene set selection

Two different gene sets were selected, one for the hematologic malignancy cohort and one for the solid tumor cohort. The sets were selected by combining available clinical panels for the relevant cancer types, with the inclusion of preliminary evidence genes and genes with limited available data. The solid tumor set is a pan-cancer panel, including genes associated with a wide range of malignancies, but does not include rare hematologic malignancy predisposition genes or all autosomal recessive bone marrow failure syndromes. The hematologic malignancy set focuses on genes associated with hematologic malignancy, immunodeficiency, and bone marrow failure. The full list of genes for each of the two sets can be found in Supplementary Table S2. Notably, the immunodeficiency genes included in the hematologic malignancy set were deliberately restricted to those with known or emerging associations specifically with hematologic malignancy predisposition or those typically included on smaller leukemia predisposition and primary immunodeficiency panels rather than all genes associated with primary immunodeficiency (of which >500 are described). This deliberate scope decision was made to maintain analytic specificity; inclusion of all immunodeficiency genes would markedly increase the denominator and dilute the analysis with genes lacking evidence for direct cancer risk.

### Matched control cohort generation

For association analyses, we generated three separate control cohorts from our full cohort of controls based on relatedness, ancestry, and sex. First, all relatives of patients with cancer were removed, leaving a control pool consisting of 1000 Genomes and birth defect Gabriella Miller Kids First Research Program (GMKF) healthy relatives. Next, a kinship coefficient (Φ) between samples was calculated using the “vcftools –relatedness2” command (21). Samples with a Φ < 0.08 were considered unrelated. Then for each cancer group (solid tumor and heme malignancy), we generated a maximally independent set of unrelated controls based on a greedy approach.

The two sets of unrelated samples were then ancestry- and sex-matched to the cases through the following procedure. A water-filling algorithm seeded the matched control population by sampling the pool of controls to match the exact case cohort ancestry–sex ratios. Then we used a greedy approach to add the remaining control samples until the matched control ancestry–sex ratios exceeded 5% of the case ratios. Control cohort sizes were determined by the available pool of unrelated, ancestry- and sex-matched controls, with the greedy sampling approach retaining as many eligible controls as possible. This yielded case-to-control ratios of approximately 1:1.8 and 1:2.75 for the hematologic malignancy and solid tumor cohorts, respectively. A formal *a priori* power calculation was not performed; however, these sample sizes are consistent with adequate power to detect moderate effect sizes in case–control association analyses. The sample sizes of the new matched controls were 2,379 for the hematologic malignancy–matched control, and 2,779 for the solid tumor–matched control. There was no significant difference in ancestry distribution between cases and matched controls [heme malignancy: *χ*^2^(4) = 4.97, *P* = 0.29; solid tumor: *χ*^2^(4) = 0.57, *P* = 0.97; *χ*^2^ test; Supplementary Fig. S3].

### Poisson rate test for rare insertion frequency analysis

To assess whether rare insertions in cancer predisposition genes occurred more frequently in cases than in matched controls, we compared insertion rates—defined as the number of insertions divided by total cohort size—using Poisson rate tests. Analyses were performed separately for the solid tumor and hematologic malignancy cohorts and were stratified by genomic location, considering insertions located in intronic, exonic, 5′ untranslated region (UTR), 3′ UTR, and all genic regions. For each genomic category, insertion rates in cases were tested against those in ancestry-matched controls. The resulting *P* values were adjusted for multiple testing using the Benjamini–Hochberg (BH) procedure.

### Alternative polyadenylation quantification

To determine functional effects of TE insertions in 3′ UTR regions, we quantified alternative polyadenylation (APA) expression using QAPA (22), a software which uses Salmon (23) to quantify expression levels for different polyadenlyation sites based on annotations from PolyASite (24).

## Results

### Landscape of germline TE insertions in nontumor genomes of patients with pediatric cancer

We analyzed germline WGS data derived from either blood or saliva from 2,894 pediatric patients with a history of cancer, collected from six cohorts from GMKF (25) and one from the DFCI PROACTIVE study (Supplementary Table S1). The GMKF program is a large NIH-supported data resource that aggregates harmonized genomic and clinical data from children with cancer and structural birth defects to enable discovery of genetic risk factors. The PROACTIVE study is a research initiative at DFCI focused on identifying inherited cancer susceptibility through comprehensive germline sequencing of oncology patients and their families. Our study examined the pediatric PROACTIVE cohort.

For controls, we analyzed 3,652 samples from healthy relatives (as defined by a lack of noted phenotypes or diagnoses) of children with birth defects from the GMKF program who had no personal history of cancer (Supplementary Table S1).

**Figure 1.**
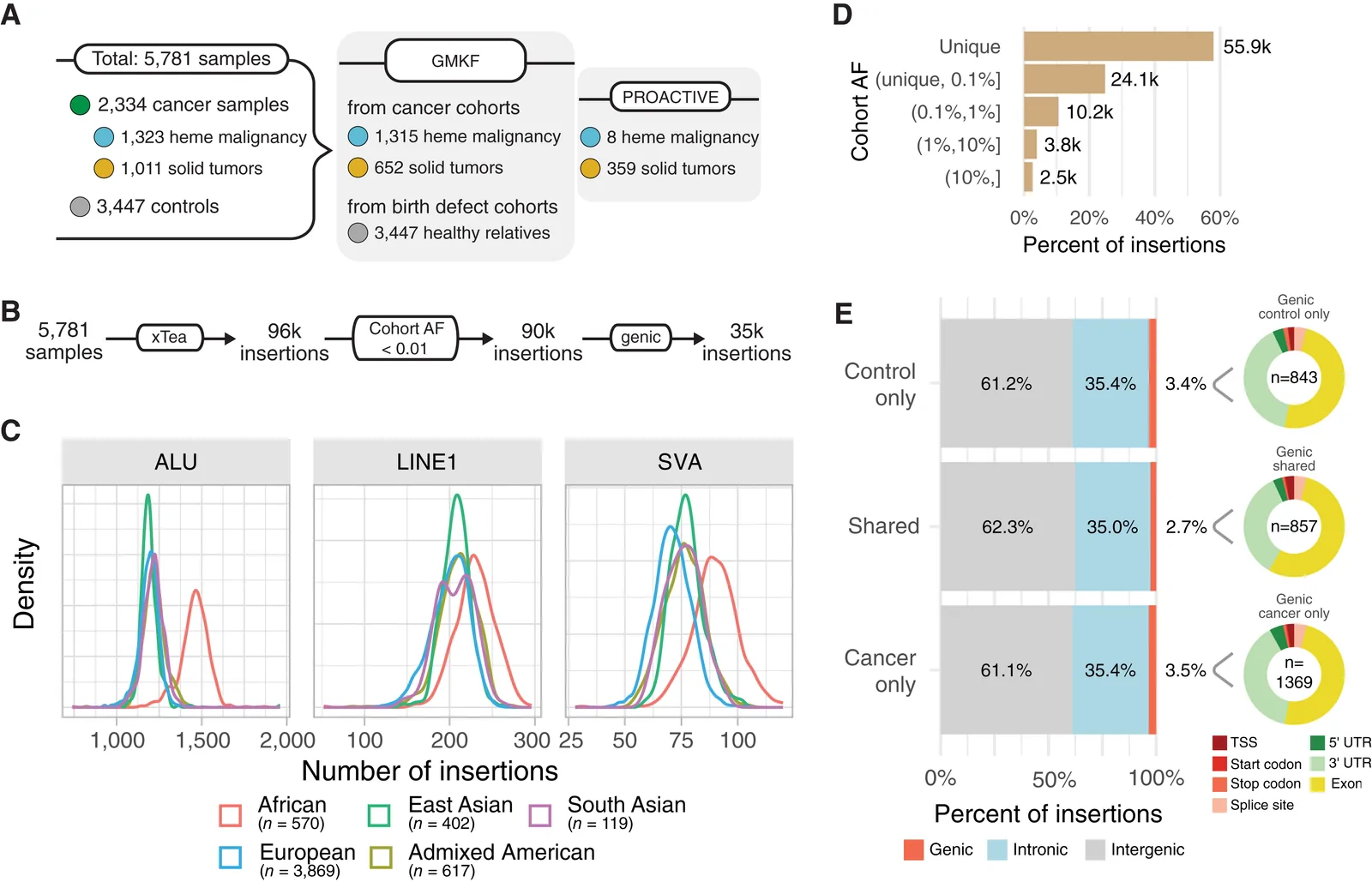
**A,** Cohorts included in the analysis. In total, we included 5,781 samples from the GMKF and the DFCI PROACTIVE study. **B,** Workflow for TE discovery and filtering. Using xTea, 96,000 TE insertions were identified and filtered by cohort AF and genic context, yielding 35,000 candidates. **C,** Density distributions of per-individual TE counts, stratified by ancestry and TE type. **D,** Distribution of cohort AF across TE insertions. **E,** Genomic distribution of TE insertions across intronic, intergenic, and genic annotations; genic annotations are further subdivided into transcription start site (TSS), start/stop codons, splice sites, 5′ and 3′ UTRs, and exons.

After quality control (see “Materials and Methods”; Supplementary Fig. S1), we retained 2,334 of 2,894 patients with cancer and 3,447 controls for a total of 5,781 for further analyses (Fig. 1A). The cancer cases consisted of 1,323 patients with a hematologic malignancy and 1,011 patients with a solid tumor or a central nervous system (CNS) tumor. Within each cohort, a wide range of pediatric cancer diagnoses were represented, with T-cell acute lymphoblastic leukemia (T-ALL) representing 96% of the hematologic malignancy cohort and neuroblastoma (44%), germ cell tumors (13.5%), and Ewing sarcoma (7.1%) representing the most common diagnoses in the solid tumor cohort.

We identified germline TE insertions for all 5,781 samples using xTea (cross-platform TE analyzer; ref. 16), a TE detection algorithm we developed for detecting nonreference germline TE insertions based on discordant read pairs and target-site duplication sequences characteristic of TE insertions. xTea is particularly advantageous for detection of SVAs, which have highly variable lengths and complex internal structure (26). In total, our analysis revealed 96,484 germline TE insertions encompassing Alu, L1, and SVA elements (Fig. 1B). The median nonreference insertion number per genome was 1,222 Alu, 207 L1, and 73 SVA elements, which is consistent with previous estimates (Fig. 1C; ref. 5). Median per-genome TE insertion counts varied by genetic ancestry, with individuals of African ancestry showing higher medians—1,463 Alu, 230 L1, and 90 SVA insertions per genome (Supplementary Fig. S2)—consistent with previous structural variant (SV) analysis from the 1000 Genomes project (27). We detected no significant enrichment in the number of germline TE insertions per individual in the genomes of patients with pediatric cancer as compared with controls (median number of 1,203 Alu, 199 L1, and 75 SVA insertions; Supplementary Fig. S2).

Most TE insertions were unique, with 58% observed in a single individual and 93% occurring with a combined cohort (cases and controls) AF < 1% (Fig. 1D), consistent with findings in a similar SV dataset (28). Across both cases and controls, insertion genomic location was highly similar: approximately 61% of all detected insertions were found in intergenic regions, 35% in intronic regions, and 3% to 4% in genic regions, corresponding to 857 insertions detected in both cancer and control cohorts (Fig. 1E). Additionally, the percent of individuals with a genic insertion was similar between case (82.9%) and control (83.4%) cohorts.

**Figure 2.**
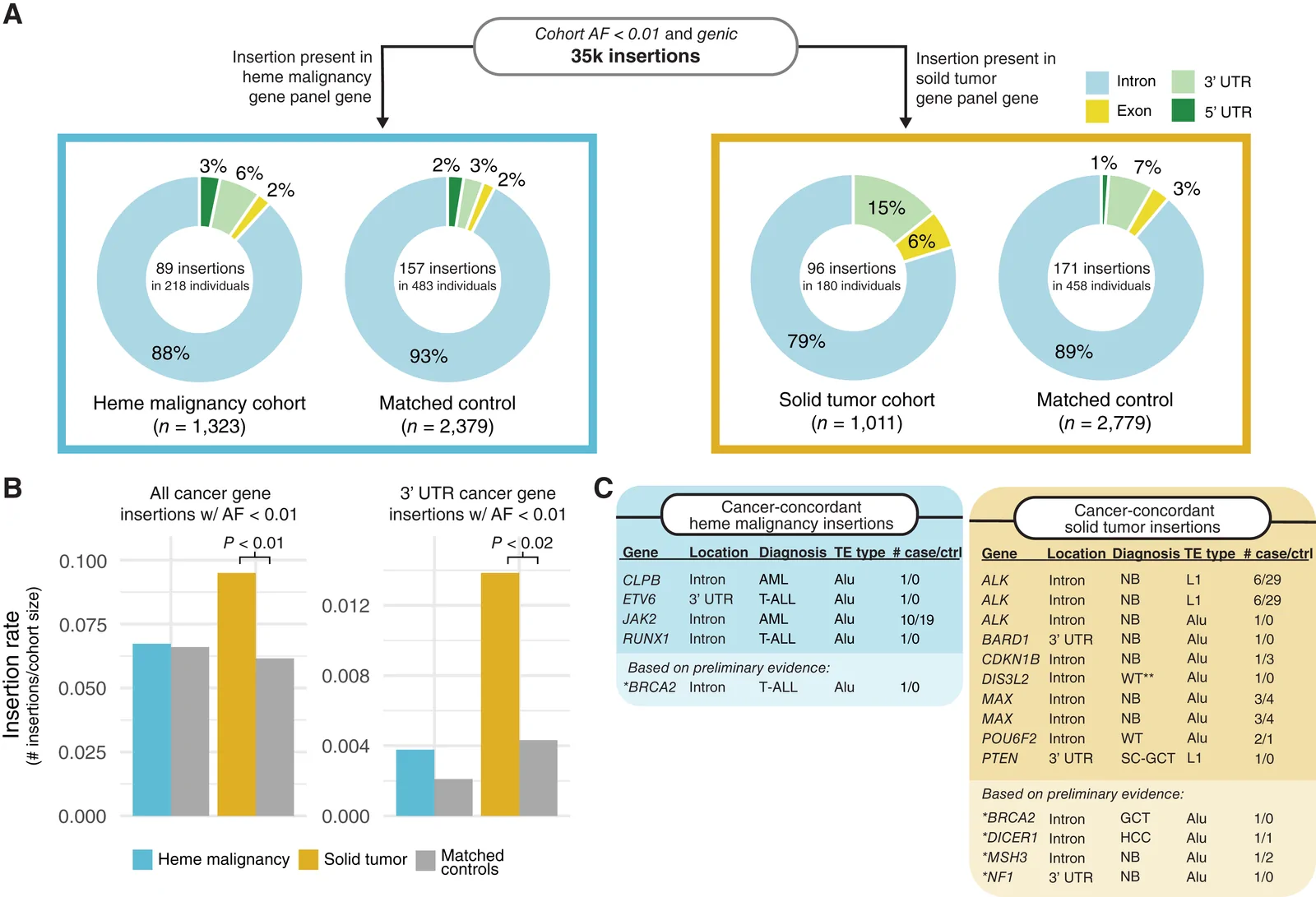
**A,** Filtering of TE insertions by cancer-subtype gene sets. Two disease-specific gene sets (hematologic malignancies; solid tumors) were used to classify insertions of interest according to each patient’s cancer subtype; donut charts depict the genic annotation distribution of relevant TE insertions. **B,** Comparison of TE insertion rates in cancer genes overall (left) and restricted to 3′ UTR insertions (right), limited to variants with cohort AF < 0.01; the solid tumor cohort shows a significantly increased insertion rate compared with a population-matched control set (Poisson rate test, BH-corrected). **C,** Nineteen TE insertions identified in genes with dominant inheritance and established associations with cancer predisposition. These constitute the highest-confidence TE insertion calls with potential pathogenic relevance. Insertions marked with (*) are considered cancer-concordant based on preliminary evidence. **The patient with WT and a *DIS3L2* intronic TE insertion was also diagnosed with Beckwith–Wiedemann Syndrome. AML, acute myeloid leukemia; GCT, germ cell tumor; HCC, hepatocellular carcinoma; NB, neuroblastoma; SC-GCT, sacrococcygeal germ cell tumor; WT, Wilms tumor.

Given the absence of global differences in TE insertion burden or distribution between cases and controls, we next focused on subsets of insertions with greater potential clinical relevance. Specifically, we prioritized rare TE insertions (cohort AF < 0.01) overlapping known or preliminary evidence cancer predisposition genes. This resulted in 583 candidate insertions across cases and controls (Fig. 2A). Subsequent analyses focused on this subset to maximize the likelihood of clinical relevance.

### Pediatric patients with solid tumors have a higher rate of rare TE insertions in 3′ UTR regions of cancer genes than controls

Next, we characterized the impact of rare TE insertions in cancer predisposition genes. To accomplish this, we compiled gene sets with established or preliminary associations to solid tumor and hematologic malignancy predisposition (see “Materials and Methods”; Supplementary Table S2). We intersected cohort TE insertions with these gene sets, applying the solid tumor set to the solid tumor cohort and the hematologic malignancy set to the hematologic malignancy cohort. For comparison, we also identified TE insertions in ancestry-matched control groups for each cohort (see “Materials and Methods”; Supplementary Fig. S3).

In the hematologic malignancy cohort, we identified 89 insertions in hematologic malignancy predisposition genes; in the solid tumor cohort, we identified 96 insertions in solid tumor predisposition genes. We observed no significant difference between cases and matched controls in the proportion of individuals harboring cancer gene insertions with AF <1%, with prevalence ranging from 16% to 20% of patients across the solid tumor, hematologic malignancy, and matched control cohorts (Supplementary Fig. S4A).

Although cases and controls showed comparable proportions of individuals with insertions, the genomic contexts in which these insertions occurred differed. For example, between 79% to 93% of rare cancer gene insertions were intronic; however, in the solid tumor cohort, more than 20% of rare insertions in cancer predisposition genes were nonintronic, including 15% in the 3′ UTR (Fig. 2A).

Additionally, within the solid tumor cohort, the frequency of rare insertions in cancer predisposition genes (number of insertions/cohort size) was significantly higher than in matched controls (*P* < 0.01, Poisson rate test, BH-corrected, see “Materials and Methods”). This pattern held for the rate of rare insertions in 3′ UTRs of cancer predisposition genes and nonintronic regions, which were likewise elevated relative to matched controls (*P* < 0.02 and *P* < 0.01 respectively, Poisson rate test, BH-corrected; Fig. 2B). This effect remains significant when restricting analysis to only GMKF cancer cohorts, ensuring the robustness of the result regardless of possible batch effects (Supplementary Fig. S4B). We also observed that the exonic and intronic insertion rate was similarly elevated in the solid tumor cohort but not at a level reaching significance (Supplementary Fig. S4C). The hematologic malignancy cohort similarly showed a trend toward a higher frequency of rare insertions in the 3′ UTRs of associated cancer predisposition genes but without reaching statistical significance.

Next, we evaluated gene–phenotype concordance to see whether individuals with TE insertions in cancer predisposition genes had developed tumor types with known associations to those genes (cancer-concordant insertions). We restricted this analysis to insertions in genes associated with autosomal dominant risk. In total, we found 19 insertions in cancer-concordant genes, which corresponds to 0.81% of samples in our total cancer cohort (1.4% of the solid tumor cohort and 0.38% of the hematologic malignancy cohort; Fig. 2C). We also confirmed that no relevant SNVs for these individuals were labeled as pathogenic or likely pathogenic in ClinVar that might otherwise explain their cancer phenotype. One patient who had a dominant cancer-concordant TE insertion was also found to have a phenotype relevant co-occurring condition: a *DIS3L2* intronic TE insertion in a patient with Wilms tumor was also diagnosed with Beckwith–Wiedemann syndrome.

### Relevant TE insertions disrupt RNA transcripts

To investigate the functional effect of insertions with high likelihood of pathogenicity, we analyzed RNA-seq data for rare TE insertions (cohort AF < 0.01) that were present in genes associated with solid tumors or hematologic malignancies. We examined GMKF samples for which tumor RNA-seq data were available and PROACTIVE samples for which peripheral blood mononuclear cell (PBMC) RNA-seq data were available. This yielded RNA-seq data for 182 of the 327 insertions (among 157 of 280 patients).

We first manually validated each of the 182 insertion calls by visually inspecting the pattern of WGS reads in the neighborhood of the corresponding genomic loci. Then we investigated the presence of TE-derived reads in matched RNA-seq data. Overall, we found that 30% (55 of 182) of RNA-seq samples showed at least one read containing the inserted TE sequence. Notably, of the 13 insertions located in the 3′ UTR with RNA-seq available, 61% (8 of 13) showed evidence of transcription of the inserted TE sequences. In cases with no evidence of transcripts containing the TE sequence, it may be due to low expression of the gene (at least for the given RNA-seq depth) or the absence of intronic insertions from mature transcripts.

**Figure 3.**
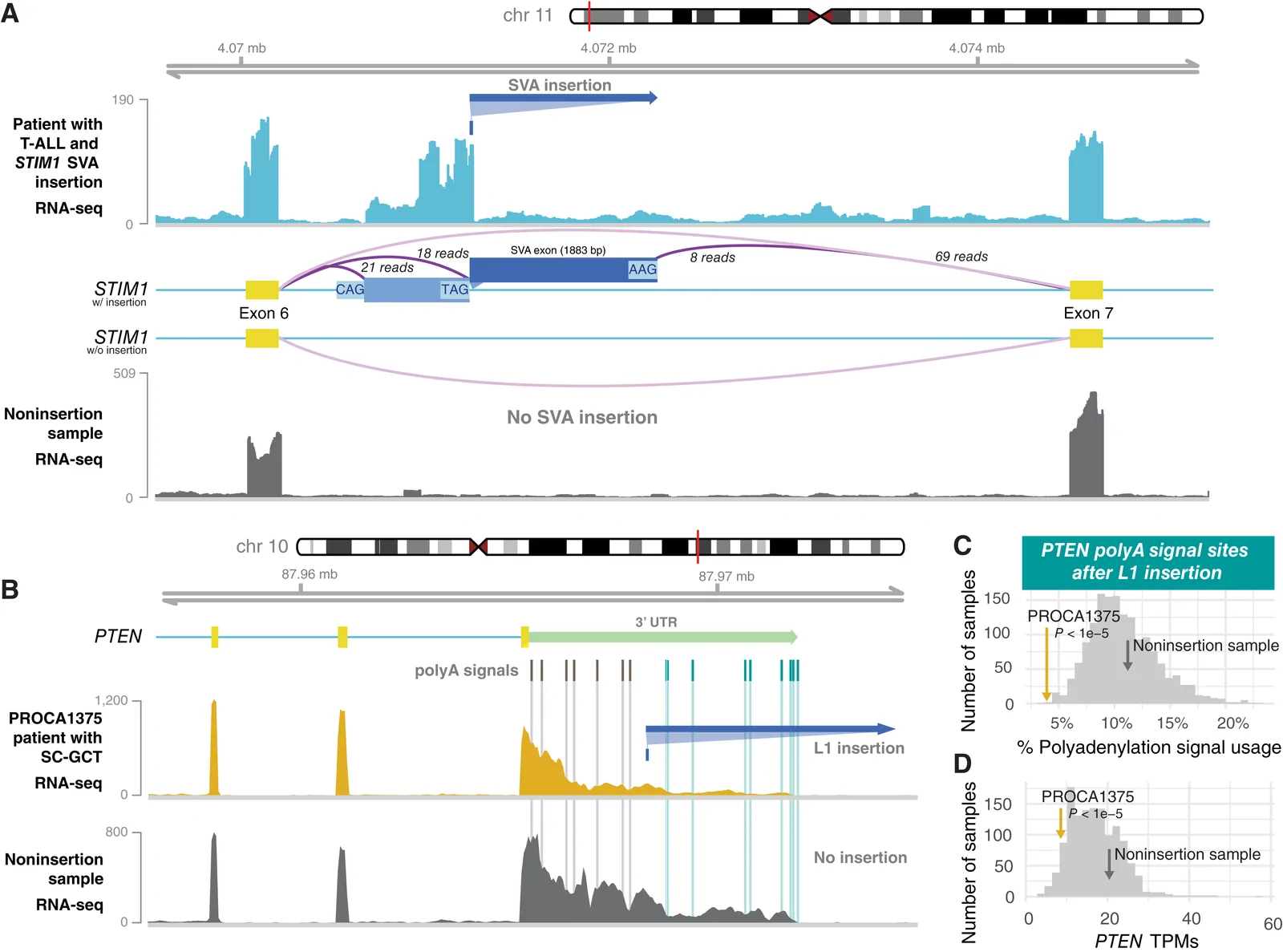
**A,** RNA-seq coverage at the *STIM1* locus for a patient harboring an SVA-driven exonization and for a control; purple arcs indicate splice-junction reads detected in the patient (top) and control (bottom). **B,** RNA-seq coverage of the *PTEN* 3′ UTR in PROCA1375 (harboring a L1 insertion) vs. a control sample; vertical lines mark canonical PTEN polyadenylation sites (PAS), with teal denoting PAS located downstream of the L1 insertion. **C,** Distribution of the summed percent usage across the nine PAS downstream of the L1 insertion across 1,599 PROACTIVE RNA-seq samples; the yellow arrow marks PROCA1375, which shows significantly reduced PAS usage (*t* test, *P* < 1 × 10^−5^), and the gray arrow marks the control sample shown in **B**. **D,** Distribution of *PTEN* expression (TPM) across 1,599 PROACTIVE RNA-seq samples; yellow and gray arrows indicate PROCA1375 and the control sample, respectively.

Among these insertions, we identified an exonization event (acquisition of a new exon) in a patient with T-ALL harboring a heterozygous SVA insertion in an intron of *STIM1* (Fig. 3A). This insertion was not observed in any other individual in the entire cohort. Analysis of tumor RNA-seq data revealed aberrant splicing patterns between exons 6 and 7 of *STIM1*, consistent with the SVA activating two cryptic splice sites and introducing a novel SVA-based exon (Fig. 3A). Activation of cryptic splice sites and exonization by SVA elements have been observed in several cases previously (29). Although *STIM1* has not been previously implicated in germline predisposition to ALL, it controls multiple proinflammatory pathways in leukemia T lymphoblasts (30). Collectively, these findings provide direct evidence of an intronic SVA insertion that drives exonization and aberrant splicing of *STIM1*, with potential functional consequence.

Additionally, we identified a unique, full-length L1 insertion in the 3′ UTR of the tumor-suppressor gene *PTEN* in a patient with a sacrococcygeal germ cell tumor (PROCA1375; Fig. 3B). No additional features of PTEN hamartoma tumor syndrome (PHTS) such as macrocephaly, mucocutaneous lesions, or neurodevelopmental findings were documented in the participant’s medical record; however, they did not have a targeted exam to assess for these features. Similar to other 3′ UTR insertions, we observed many TE-derived RNA-seq reads in the patient’s blood sample. To assess the functional effect of the insertion, we quantified alternative polyadenylation usage of *PTEN*, which contains 16 known polyadenylation sites, with the L1 insertion located downstream of the seventh site. We evaluated *PTEN* alternative polyadenylation across 1,560 PBMC RNA-seq samples from the larger PROACTIVE cohort (which includes adults and children with cancer) to increase the sample size for this analysis. Specifically, we compared PROCA1375 with the rest of the cohort by summing the percent of the polyadenylation usage of the 9 polyA sites which are found after the insertion. This analysis showed that PROCA1375 exhibited significantly different polyA usage from the other PROACTIVE samples—usage at poly(A) sites downstream of the L1 insertion was significantly reduced (*P* < 1e−5, *t* test; Fig. 3C), suggesting a loss of longer transcripts. In addition, PROCA1375 had significantly lower *PTEN* transcript abundance [measured in transcripts per million (TPM)] compared with the rest of the PROACTIVE cohort (Fig. 3D). Together, these observations suggest that the L1 insertion in the 3′ UTR of *PTEN* disrupts longer transcript isoforms, impairing its expression.

## Discussion

To facilitate early detection and cancer interception for children at risk of developing cancer, there is great interest in expanding our understanding of the underlying mechanisms of germline predisposition beyond SNVs and deletions/duplications in known cancer predisposition genes. As clinical genome sequencing becomes more accessible, we need a deeper understanding of how the entire genome contributes to predisposition. Repetitive sequences make up the majority of the noncoding genome, of which TEs are the major contributor of genetic diversity in these regions. Our study provides evidence that germline TE insertions represent an underrecognized and clinically actionable source of cancer predisposition.

Several findings have important implications for how we approach genetic testing and counseling in pediatric oncology. First, the enrichment of rare TE insertions in the 3′ UTRs of cancer predisposition genes in solid tumor patients raises the possibility that germline predisposition is being missed by current testing paradigms that do not assess noncoding and repetitive regions. Similarly, the functional evidence of impaired *PTEN* expression in a patient with a sacrococcygeal germ cell tumor suggests an association with PHTS and raises the possibility that TE insertions could explain part of the missing heritability for patients with clinical diagnoses of a cancer predisposition and negative molecular testing. PHTS is associated with a broad tumor spectrum, and underrecognition has real consequences for surveillance and prevention. Despite the limited available clinical information, the absence of reported classic features would not rule out the possibility of PHTS given the broad phenotypic spectrum and variable expressivity in PHTS. These findings argue for routine inclusion of TE detection in germline sequencing workflows, particularly for patients with clinical suspicion of hereditary predisposition and uninformative standard testing. Although the role of TE insertions in tumorigenesis is emerging in the context of multiple tumor types (31), the role that these repetitive elements may play in predisposing children to cancer has not been systematically explored.

In this study, we took advantage of large, annotated datasets of germline WGS from patients with pediatric cancer to explore the role of TE insertions in pediatric cancer predisposition. We compared insertions in the germline genomes of more than 2,300 patients with pediatric cancer with a wide range of malignancies to more than 3,000 controls without cancer. Rather than a relative increase in global retrotransposition activity, we noted a distinct difference in the regional distribution of rare TE insertions between cases compared with controls, suggesting that the location of these insertions in genes implicated in cancer predisposition may be particularly important. It is important to note that the case cohort is not fully representative of the broader pediatric cancer population. T-ALL comprises 96% of our hematologic malignancy cohort, whereas B-cell ALL is the most common pediatric hematologic malignancy in the general population. In our solid tumor cohort, the most common malignancies are neuroblastoma, germ cell tumors, and Ewing sarcoma, whereas CNS tumors are the most common pediatric solid tumors in the general population. Findings should therefore be interpreted in the context of these specific cancer types, and future studies in more representative cohorts, particularly those enriched for cancers with strong germline predisposition and familial risk, are warranted.

After filtering, we found 583 rare TE insertions within cancer genes. The vast majority of these were located in intronic regions for which pathogenicity is difficult to interpret. However, there is precedent for pathogenic intronic TE insertions, for example, in aberrant splicing due to an SVA insertion (32) and inverted-repeat matching of Alus (33). We identified 9 intronic TE insertions that are good candidates for cancer predisposition, as they occur in genes with established associations with the corresponding tumor types in the heterozygous state. These include Alu insertions in *RUNX1* for a patient with T-ALL and in *ALK* for a patient with neuroblastoma.

Rare TE insertions were enriched in nonintronic areas of the genome in cases, in particular in the 3′ UTRs of cancer predisposition genes. This difference was most evident in the cohort of pediatric patients with solid tumors. Almost 15% (14/96) of low-frequency TE insertions affecting cancer predisposition genes in the solid tumor cohort were located in 3′ UTRs, including two insertions in cancer-concordant genes (*BARD1* and *PTEN*) and one insertion in a gene with preliminary cancer-concordant evidence (*NF1*).

The hematologic malignancy cohort did not show a significant difference in the rate of TE insertions compared with the matched control cohort; we postulate that this reflects several factors: (i) the narrower spectrum of genes with strong evidence for germline predisposition to hematologic malignancies, (ii) the use of a broad gene list for this cohort, including many genes with limited or preliminary evidence for hematologic malignancy risk, and (iii) the inclusion of genes associated with myeloid malignancies and autosomal recessive inheritance. Together, these factors may have diluted the observed effect size.

The availability of RNA-seq data for a subset of patients allowed us to begin to characterize the functional consequence of TE insertions for these patients, highlighting the benefits of RNA-seq in interpreting genomic data, as well as the role that these insertions may play.

First, a patient with a sacrococcygeal germ cell tumor had a unique full-length L1 insertion in the 3′ UTR of the tumor-suppressor gene *PTEN*. We investigated RNA-seq for this patient and found significantly lower TPMs and loss of polyA site usage after the L1 insertion compared with 1,559 other samples. Interestingly in mice, the knockdown of longer *Pten* transcripts (specifically 3.3 kb+ after the last coding exon) correlates with loss of protein regardless of shorter transcript expression (34). Overall, we hypothesize that this loss of longer transcripts could lead to a decrease in *PTEN* protein production. As 3′ UTRs are not part of the translated transcript, they are likely more tolerable in the germline. However, their functional effect across the lifespan of an individual could lead to increased cancer susceptibility. Insertions in the 3′ UTR would be expected to affect mRNA stability, cellular localization, and translational efficiency that can alter tumor-suppressive functions during cellular division in different tissue contexts (35).

Second, we demonstrate that a patient with T-ALL with a SVA insertion found in an intron in *STIM1* had acquisition of a new exon due to the insertion. Tumor RNA demonstrated aberrant splicing with the activation of two cryptic splice sites near the location of the insertion. Even though *STIM1* is not thought to be the driver of this patient’s leukemia, this case clearly illustrates the ability of TE insertions to alter the coding function of genes through splicing defects. Cryptic exons in *BRCA2* have been reported in familial breast cancer families (36), and similar events from TEs can be a new mechanism for increased hereditary cancer risk. Importantly, aberrant splicing similarly caused by an SVA insertion has been shown to be therapeutically targetable with the application of antisense oligonucleotide therapeutics to restore normal gene expression (37). This highlights the potential clinical utility of identifying splice-disrupting TE insertions.

**Figure 4.**
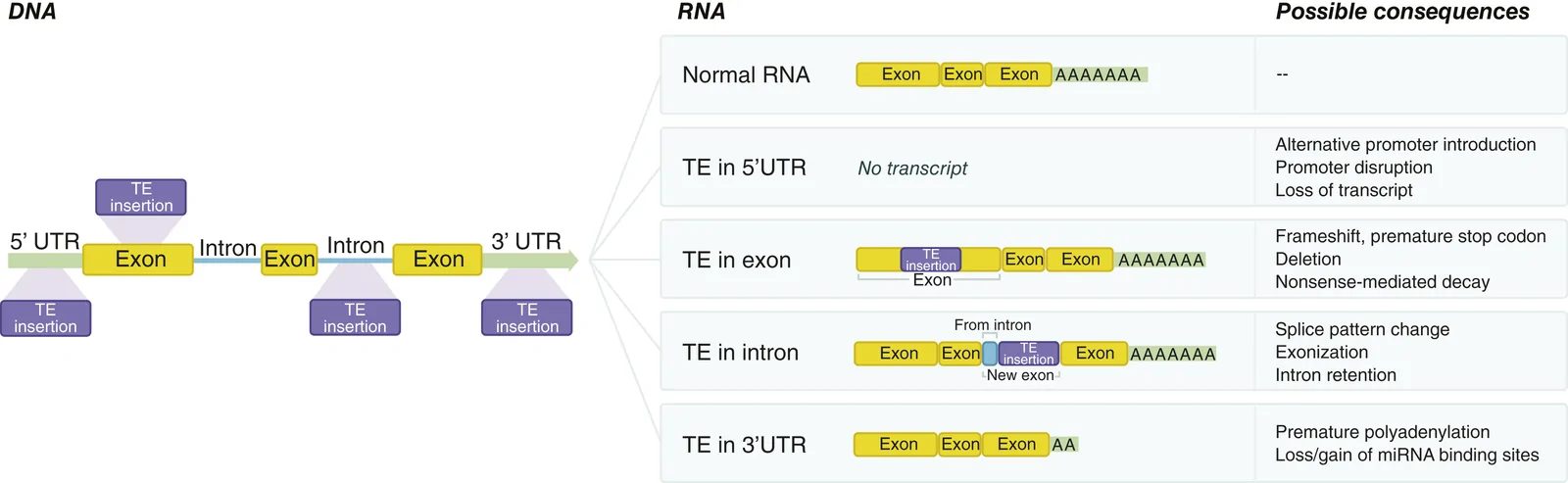
Potential functional consequences of TE insertions. The RNA panel (middle) shows the theoretical effects of the TE insertion on the RNA transcript.

Although these examples provide evidence for the potential functional consequences of insertions (Fig. 4), our study was limited by the availability of RNA-seq for only a subset of patients, restricting our ability to fully characterize the downstream significance of all observed insertion events. This may have led to an underrecognition of clinically significant insertions. Detection of TEs is also highly sensitive to sequencing data quality, and although we attempt to account for low quality, batch effects across so many distinct datasets may contribute to our results, limiting our ability to detect case–control differences and to eliminate all artifacts. Although the GMKF dataset provided a robust resource for WGS, we were also limited in our ability to look across a more representative spectrum of cancer phenotypes. The most commonly represented cancers in these datasets have only modest associations with cancer predisposition, including neuroblastoma and Ewing sarcoma in the solid tumor cohort and T-ALL in the hematologic malignancy cohort. Additionally, in some settings, there is a known association of cancer predisposition with birth defects (38). The control cohort used in our analysis, though documented as unaffected in the limited clinical data available, may carry some cancer predisposition risk given their familial association with a child with known birth defects, which could confound case–control comparisons.

TE insertions, although rare, constitute an underrecognized source of genetic variation relevant to cancer predisposition risk among children. This mechanism should also be considered for cases in which there is a clinical suspicion for a germline predisposition but for which current clinical germline testing otherwise fails to identify a cause. TE insertions should be considered and included among research efforts to uncover missing sources of heritability for patients with pediatric cancer. Even within this cohort not enriched for cancers with strong association with predisposition, we found compelling evidence for the role of TE insertions in children with cancer. Further investigation within cohorts with high suspicion for predisposition without identified germline risk factors should be examined.

## Supplementary Material

Table S1 Origin of datasets and QC filtering.

Table S2 Gene lists used for solid tumor and hematologic malignancy cohorts.

Figure S1 Quality control filters for WGS samples

Figure S2 Total TE insertion counts

Figure S3 Ancestry proportions for cases and matched control used in enrichment analysis.

Figure S4 TE insertion rate in cancer genes

## Data Availability

Data for the cancer and control cohorts from the Gabriella Miller Kids First Pediatric Research Program are available through the GMKF Data Resource Portal (https://kidsfirstdrc.org/) and through dbGAP controlled access. Corresponding study codes for other analyzed datasets can be found in Supplementary Table S1. PROACTIVE DNA and RNA datasets are available upon request to the corresponding author.

## Code Availability

All related scripts and pipelines can be found at https://github.com/parklab/KidsFirst_TE_pediatric_cancer.
