# Supplementary material for: The Role of Germline Transposable Element Insertions in Pediatric Cancer Predisposition
Source: Cancer Res Commun. 2026 Sep 22;6(9):2232–41. doi: 10.1158/2767-9764.CRC-26-0229 (PMC13595835; doi:10.1158/2767-9764.CRC-26-0229)
Supplement: Supplementary file 1 [file crc-26-0229_figure_s1_suppsf1.pdf]

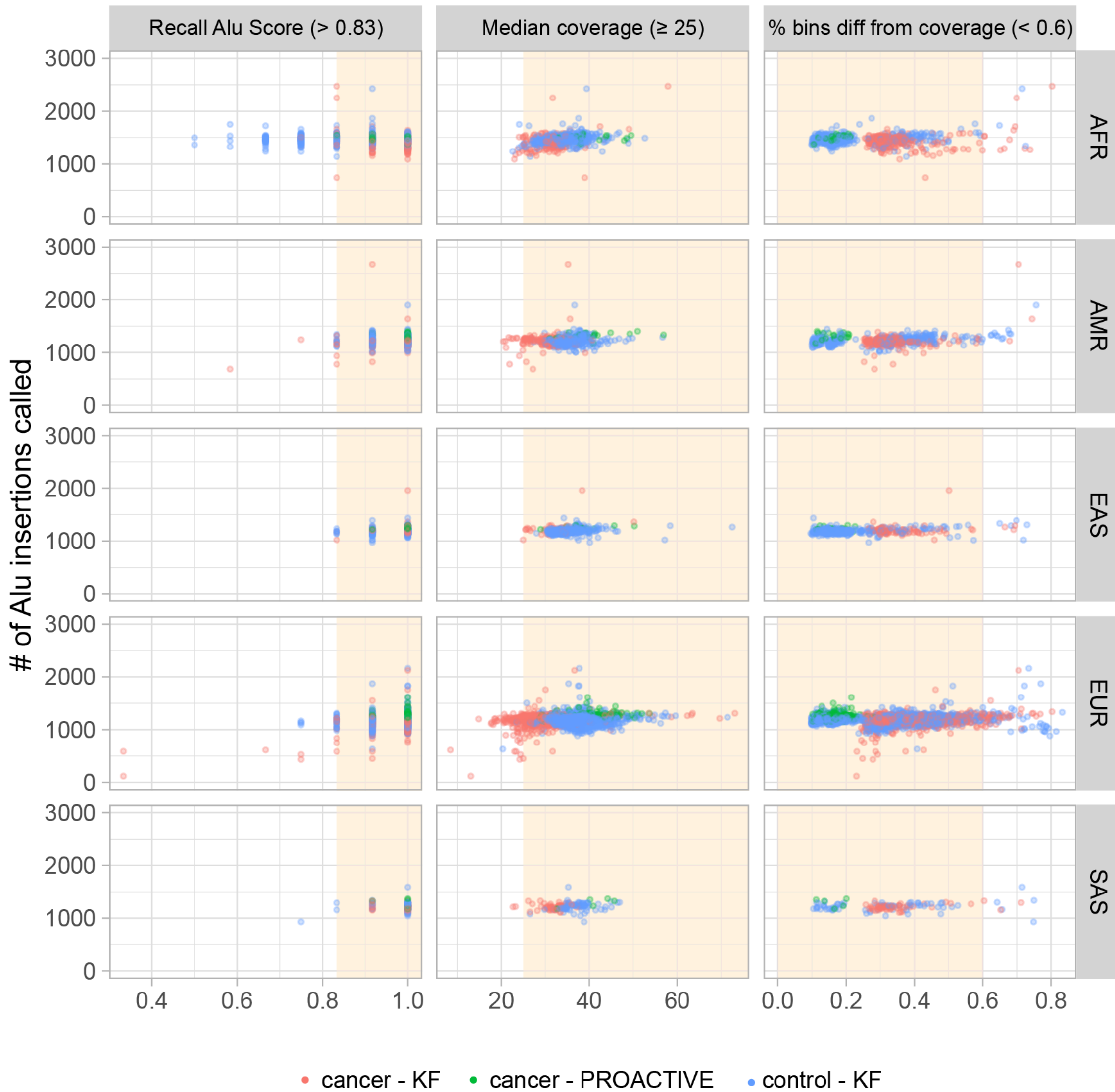

Figure S1. Quality control filters for WGS samples. The red shaded region in each facet indicates thresholds for passing QC. In total 5,781/6,546 (88%) of samples passed QC filtering. Briefly, the recall Alu score corresponds to the % of common Alu (1KG pop AF > 0.95, n = 12) insertions identified. The median coverage is the WGS coverage per sample. The % bins diff from coverage refers to the uniformity of coverage in the sample. See Methods.
