# Supplementary material for: The Role of Germline Transposable Element Insertions in Pediatric Cancer Predisposition
Source: Cancer Res Commun. 2026 Sep 22;6(9):2232–41. doi: 10.1158/2767-9764.CRC-26-0229 (PMC13595835; doi:10.1158/2767-9764.CRC-26-0229)
Supplement: Supplementary file 2 [file crc-26-0229_figure_s2_suppsf2.pdf]

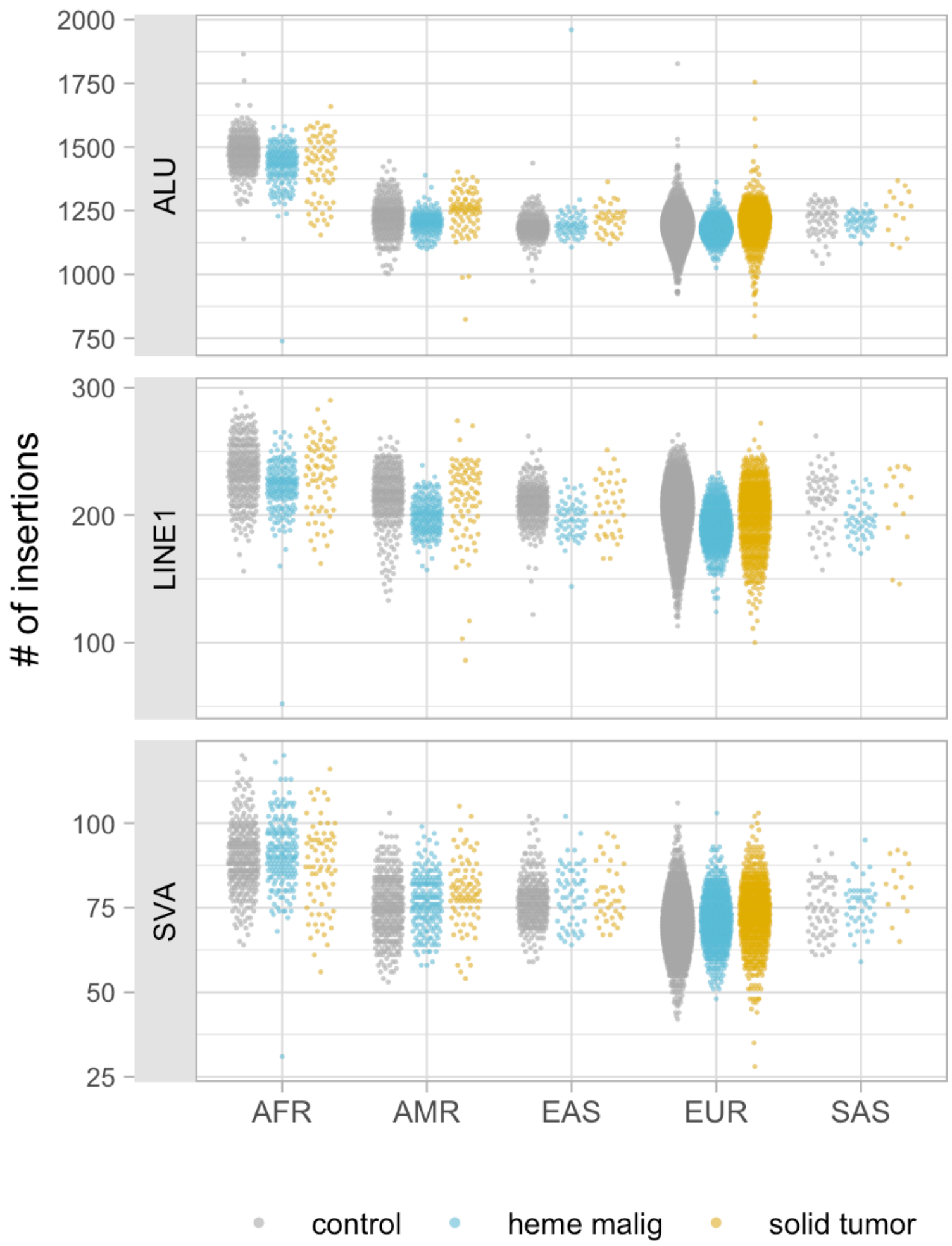

Fig S2. Total TE insertion counts for all 5,781 cases and controls separated by population and cohorts.
