# Supplementary material for: The Role of Germline Transposable Element Insertions in Pediatric Cancer Predisposition
Source: Cancer Res Commun. 2026 Sep 22;6(9):2232–41. doi: 10.1158/2767-9764.CRC-26-0229 (PMC13595835; doi:10.1158/2767-9764.CRC-26-0229)
Supplement: Supplementary file 3 [file crc-26-0229_figure_s3_suppsf3.pdf]

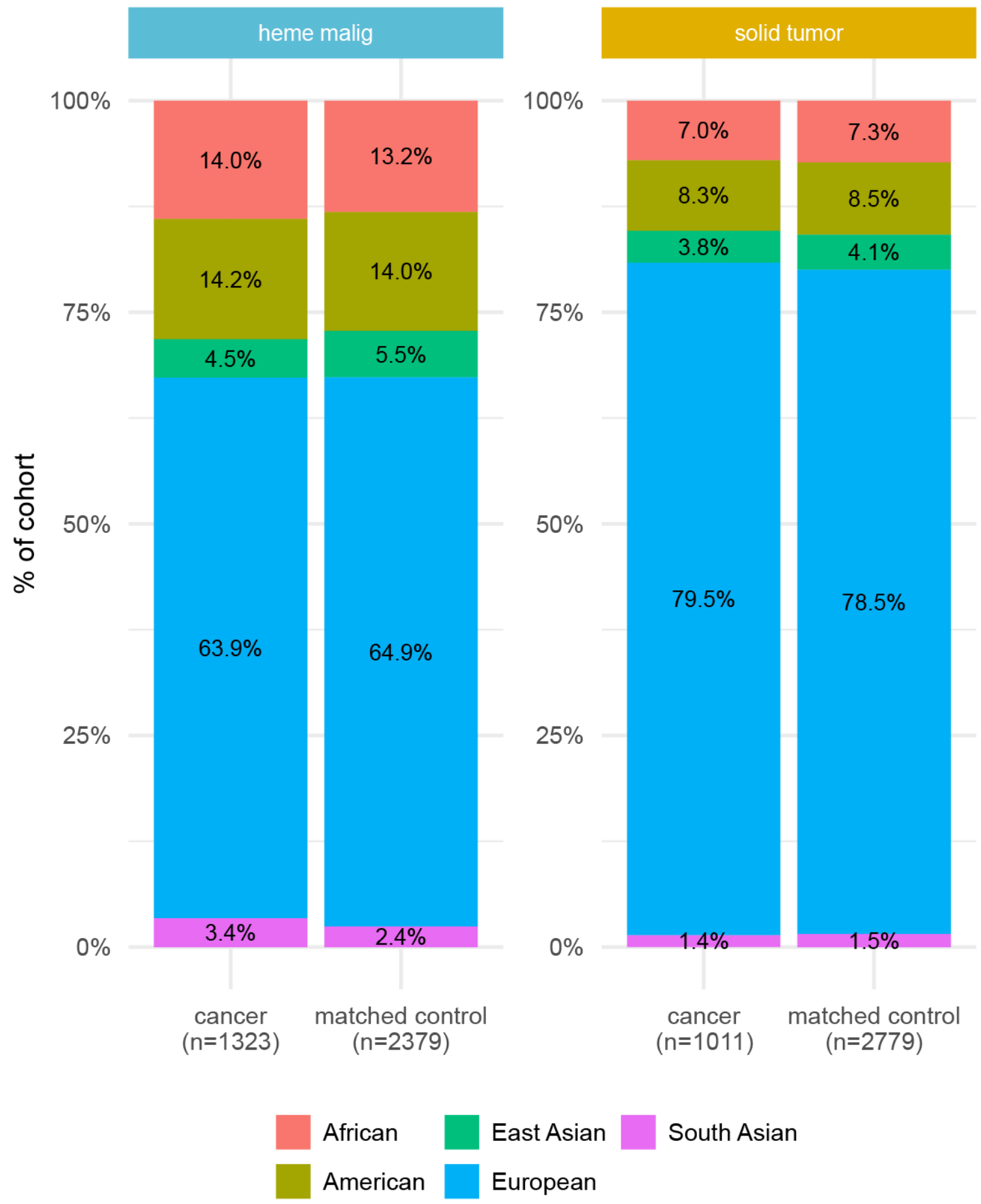

Figure S3. Ancestry proportions for cases and matched control used in enrichment analysis. There was no significant difference in ancestry distribution between cases and controls (heme malignancy: $\chi^2(4) = 4.97$, $p = 0.29$; solid tumor: $\chi^2(4) = 0.57$, $p = 0.97$; chi-square test).
