# Supplementary material for: The Role of Germline Transposable Element Insertions in Pediatric Cancer Predisposition
Source: Cancer Res Commun. 2026 Sep 22;6(9):2232–41. doi: 10.1158/2767-9764.CRC-26-0229 (PMC13595835; doi:10.1158/2767-9764.CRC-26-0229)
Supplement: Supplementary file 4 [file crc-26-0229_figure_s4_suppsf4.pdf]

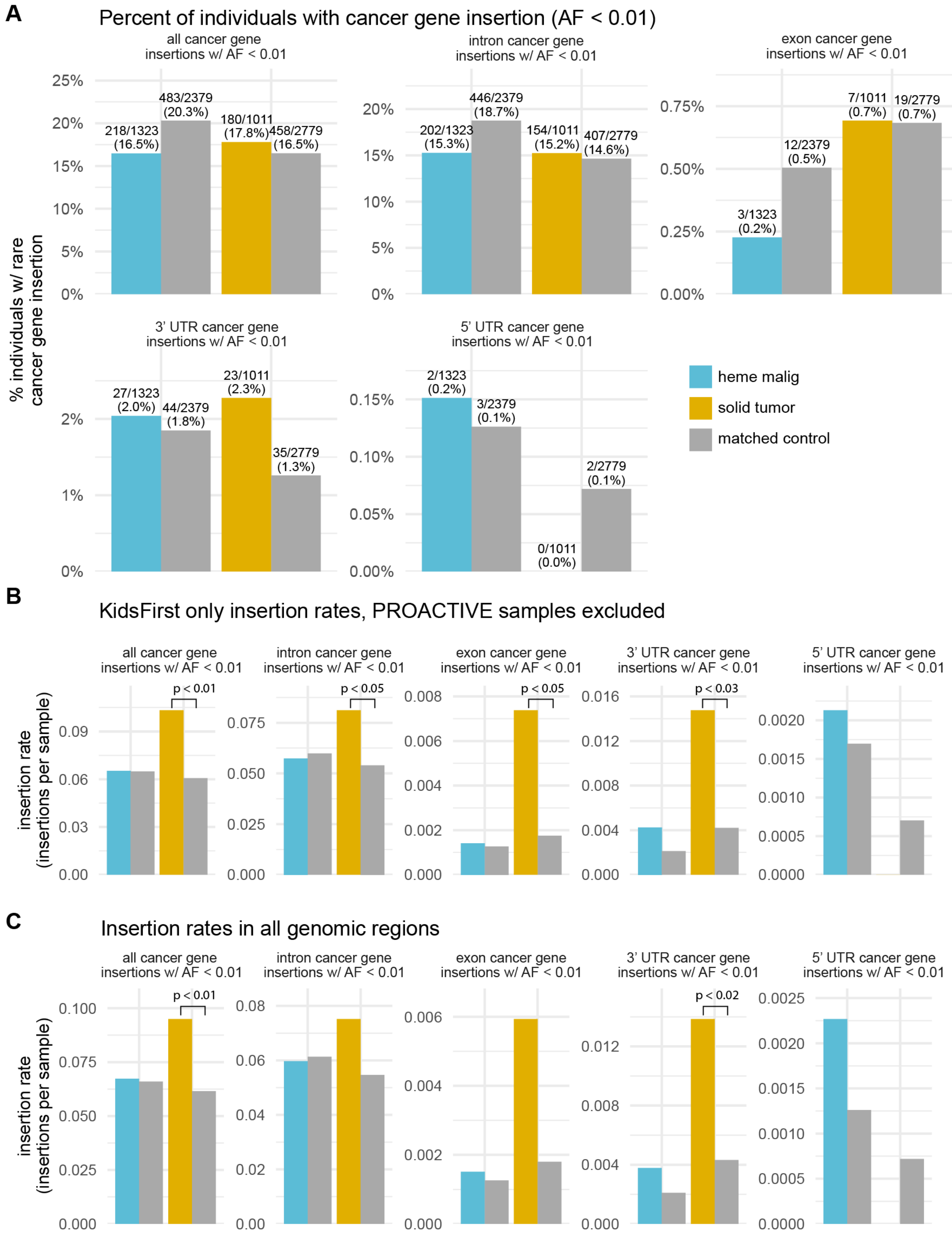

Figure S4. (A) The percent of individuals in each cohort with a rare (<1% AF) cancer gene insertion based on filtering by cancer-type specific gene panels. None of these comparisons reach significance by Fisher's exact test. (B) Insertion rates for TEs in cancer genes with rare AF subset to exclude PROACTIVE dataset. Significant 3'UTR findings are robust to dataset batch effects. (C) Insertion rates for TEs in cancer genes with rare AF, all regions shown. While only "3'UTR" and "all" categories are significant by Poisson rate test, moderate enrichment in the solid tumor cohort is seen in introns and exons, but not at a level reaching significance.
