# Supplementary material for: The Role of Germline Transposable Element Insertions in Pediatric Cancer Predisposition
Source: Cancer Res Commun. 2026 Sep 22;6(9):2232–41. doi: 10.1158/2767-9764.CRC-26-0229 (PMC13595835; doi:10.1158/2767-9764.CRC-26-0229)
Supplement: Supplementary file 5 [file crc-26-0229_table_s1_suppst1.pdf]

Table S1. Origin of datasets and QC filtering.

Study Name	Study Code	dbGaP accession	# Cases	# Controls	Total Samples	# samples pass QC	% samples pass QC
**CASES**							
PROACTIVE	PROACTIVE		381	0	381	367	96.33%
Kids First: Ewing Sarcoma – Genetic Risk	KF-ESGR	phs001228	400	0	400	62	15.50%
Kids First: Genetic Predisposition to Intracranial Germ Cell Tumors	KF-IGCT	phs002322	132	0	132	128	96.97%
Kids First: Germline and Somatic Variants in Myeloid Malignancies in Children	KF-MMC	phs002187	38	0	38	37	97.37%
Discovering the Genetic Basis of Human Neuroblastoma: A Gabriella Miller Kids First Pediatric Research Program (Kids First) Project	KF-NBL	phs001436	559	0	559	384	68.69%
Gabriella Miller Kids First Pediatric Research Program in Novel Cancer Susceptibility in Families (from BASIC3)	KF-NCSF	phs001683	81	0	81	78	96.30%
Comprehensive Genomic Profiling to Improve Prediction of Clinical Outcome for Children with T-cell Acute Lymphoblastic Leukemia	KF-TALL	phs002276	1,303	0	1,303	1,278	98.08%
**CONTROLS**							
Kids First: Whole genome sequencing of nonsyndromic craniosynostosis	KF-NC	phs001806	0	476	476	435	91.39%
Whole Genome Sequencing of African and Asian Orofacial Clefts Case-Parent Triads	KF-OFCAA	phs001997	0	472	472	378	80.08%
Kids First: Genomics of Orthopaedic Disease Program	KF-AIS	phs001410	0	272	272	260	95.59%
Kids First: Genomic Analysis of Congenital Diaphragmatic Hernia	KF-CDH	phs001110	0	983	983	976	99.29%
National Heart, Lung, and Blood Institute (NHLBI) Bench to Bassinet Program: The Gabriella Miller Kids First Pediatric Research Program of the Pediatric Cardiac Genetics Consortium (PCGC)	KF-CHD	phs001138	0	1,305	1,305	1,286	98.54%
Kids First: Genetic Basis of Disorders/Differences of Sex Development (DSD)	KF-DSD	phs001178	0	144	144	112	77.78%
	**TOTALS:**		**2,894**	**3,652**	**6,546**	**5,781**	**88.31%**
