# Supplementary material for: The Role of Germline Transposable Element Insertions in Pediatric Cancer Predisposition
Source: Cancer Res Commun. 2026 Sep 22;6(9):2232–41. doi: 10.1158/2767-9764.CRC-26-0229 (PMC13595835; doi:10.1158/2767-9764.CRC-26-0229)
Supplement: Supplementary file 6 [file crc-26-0229_table_s2_suppst2.pdf]

Table S2. Gene lists used for solid tumor and hematologic malignancy cohorts.

SOLID TUMOR GENE LIST (N=194)		
ABRAXAS1	FANCM	RAD51
ACD	FBXW7	RAD51C
AIP	FH	RAD51D
AKT1	FLCN	RB1
ALK	FOCAD	RECQL
AMER1	GALNT12	RECQL4
AP2S1	GATA1	REST
APC	GATA2	RET
ARID1A	GCM2	RHBDF2
ASXL1	GEN1	RINT1
ATM	GNA11	RNF43
ATR	GPC3	RPL11
AXIN2	GPC4	RPL15
BAP1	GPR161	RPL19
BARD1	GREM1	RPL26
BLM	HACE1	RPL35A
BMPR1A	HNF1A	RPL5
BRCA1	HNF1B	RPS10
BRCA2	HOXB13	RPS19
BRIP1	HRAS	RPS20
BUB1	IGF2	RPS24
BUB1B	KDM3B	RPS26
CASR	KIF1B	RPS29
CDC73	KIT	RPS7
CDH1	LZTR1	RTEL1
CDK4	MAX	RUNX1
CDKN1B	MBD4	SDHA
CDKN1C	MC1R	SDHAF2
CDKN2A	MDH2	SDHB
CEBPA	MDM2	SDHC
CEP57	MEN1	SDHD
CFTR	MET	SLC25A11
CHEK2	MITF	SLX4
CPA1	MLH1	SMAD4
CTC1	MLH3	SMARCA4
CTNNA1	MPL	SMARCB1
CTR9	MRE11	SMARCE1
CTRC	MSH2	SPINK1
CYLD	MSH3	SPRED1
DGCR8	MSH6	STK11
DICER1	MUTYH	SUCLG2
DIS3L2	NBN	SUFU
DKC1	NF1	TERC
DLST	NF2	TERT
DROSHA	NHP2	TINF2
EGFR	NOP10	TMEM127
EGLN1	NTHL1	TP53
ELANE	NYNRIN	TRIM28
ELP1	PALB2	TRIM37
ENG	PALLD	TRIP13
EPAS1	PARN	TRPV6
EPCAM	PDGFRA	TSC1
ERBB2	PHOX2B	TSC2
ERCC4	PIK3CA	USB1
EXT1	PMS2	USP45
EXT2	POLD1	VHL
EZH2	POLE	WAS
FANCA	POT1	WRAP53
FANCB	POU6F2	WRN
FANCC	PPM1D	WT1
FANCD2	PRKAR1A	XPO5
FANCE	PRSS1	XRCC2
FANCF	PTCH1	
FANCG	PTCH2	
FANCI	PTEN	
FANCL	RAD50	

HEMATOLOGIC MALIG GENE LIST (N=194)		
ABCB7	HPS5	RPS10
ACD	HPS6	RPS15A
ADA2	HTRA2	RPS17
AK2	HYOU1	RPS19
ALAS2	IFNGR2	RPS20
ANKRD26	IKZF1	RPS24
AP3B1	ITGA2B	RPS26
ATM	ITK	RPS27
ATR	JAGN1	RPS28
ATRX	JAK2	RPS29
BLM	KCNN4	RPS7
BLOC1S3	KDM1A	RTEL1
BLOC1S6	KLF1	RUNX1
BRCA1	KRAS	SAMD14
BRCA2	LAMTOR2	SAMD9
BRIP1	LIG4	SAMD9L
CBL	LYST	SBDS
CD40	MAD2L2	SBF2
CD40LG	MAGT1	SEC23B
CDAN1	MBD4	SEPTIN6
CEBPA	MECOM	SETBP1
CEBPE	MLH1	SH2D1A
CHEK2	MPIG6B	SLC19A2
CLPB	MPL	SLC25A38
CSF3R	MSH2	SLC37A4
CTC1	MSH6	SLX4
CTLA4	MYH9	SMARCD2
CXCR4	MYSM1	SMPD1
DDX41	NAF1	SRP54
DKC1	NBN	SRP72
DNAJC21	NF1	STAT3
DNASE2	NHP2	STIM1
DNMT3A	NOP10	STK4
DTNBP1	NPM1	STN1
EFL1	NRAS	STX11
ELANE	PALB2	STXBP2
EPCAM	PARN	TAFAZZIN
EPO	PAX5	TAZ
ERCC4	PGM3	TBXAS1
ERCC6L2	PIEZO1	TCIRG1
ETV6	PMS2	TCN2
FANCA	POT1	TERC
FANCB	PRF1	TERT
FANCC	PTPN11	TET2
FANCD2	PTPRJ	THPO
FANCE	PUS1	TIMM50
FANCF	RAB27A	TINF2
FANCG	RAC2	TP53
FANCI	RAD23B	TSR2
FANCL	RAD51	TUBB1
FANCM	RAD51C	UBE2T
FAS	RBM8A	UNC13D
FASLG	RECQL4	USB1
G6PC3	RFWD3	VPS13B
GATA1	RMRP	VPS45
GATA2	RPL11	WAS
GFI1	RPL15	WDR1
GINS1	RPL17	WIPF1
GLRX5	RPL18	WRAP53
GP1BA	RPL19	XIAP
HAX1	RPL26	XRCC2
HLTF	RPL27	ZCCHC8
HOXA11	RPL31	
HPS1	RPL35	
HPS3	RPL35A	
HPS4	RPL5	
